# Matching Fishers’ Knowledge and Landing Data to Overcome Data Missing in Small-Scale Fisheries

**DOI:** 10.1371/journal.pone.0133122

**Published:** 2015-07-15

**Authors:** Ludmila de Melo Alves Damasio, Priscila F. M. Lopes, Rafael D. Guariento, Adriana R. Carvalho

**Affiliations:** 1 Graduation Program in Development and Environment at Federal University of Rio Grande do Norte/UFRN, Natal, RN, Brazil; 2 Department of Ecology at Federal University of Rio Grande do Norte/UFRN, Natal, RN, Brazil; 3 Fisheries and Food Institute, Santos, SP, Brazil; 4 Center of Biological and Health Sciences University of Mato Grosso do Sul/UFMS, Campo Grande, MS, Brazil; Aristotle University of Thessaloniki, GREECE

## Abstract

**Background:**

In small-scale fishery, information provided by fishers has been useful to complement current and past lack of knowledge on species and environment.

**Methodology:**

Through interviews, 82 fishers from the largest fishing communities on the north and south borders of a Brazilian northeastern coastal state provided estimates of the catch per unit effort (CPUE) and rank of species abundance of their main target fishes for three time points: current year (2013 at the time of the research), 10, and 20 years past. This information was contrasted to other available data sources: scientific sampling of fish landing (2013), governmental statistics (2003), and information provided by expert fishers (1993), respectively.

**Principal Findings:**

Fishers were more accurate when reporting information about their maximum CPUE for 2013, but except for three species, which they estimated accurately, fishers overestimated their mean CPUE per species. Fishers were also accurate at establishing ranks of abundance of their main target species for all periods. Fishers' beliefs that fish abundance has not changed over the last 10 years (2003–2013) were corroborated by governmental and scientific landing data.

**Conclusions:**

The comparison between official and formal landing records and fishers' perceptions revealed that fishers are accurate when reporting maximum CPUE, but not when reporting mean CPUE. Moreover, fishers are less precise the less common a species is in their catches, suggesting that they could provide better information for management purposes on their current target species.

## Introduction

Small-scale fisheries (SSFs) provide food security, welfare, and livelihood sustenance for many coastal communities in the poorest countries in the world [1]. Despite and also due to their relevance, fishing resources have been intensively exploited, causing large effects on marine ecosystems worldwide [2].

Marine and continental fisheries (including aquaculture) are estimated to have produced 154 million tons of fish worldwide in 2011 [3]. About 55 million direct jobs are generated by these activities, of which up to 90% are occupied by small-scale fishers who end up supplying half of the world's fish [4]. Brazil produced 1.4 million tons of fish in 2011, including aquaculture, marine, and continental fishing. Following the world trend, small-scale fishers catch 45% of the continental and marine fish in Brazil. Most of these fisheries are located at the northeastern coast, which alone provides 32% of the Brazilian production. The Brazilian small-scale fishing sector employs 957,000 people, showing its social and economic relevance [5].

Nevertheless, the Brazilian SSFs are highly overlooked, which also seems to be a tendency worldwide for SSFs [6] [7]. The lack of public policies for the sector results in unreported and unregulated activity around the world, for which there are barely any financial resources, staff, logistics, or skills in management agencies to collect fishery data [8]. What is known usually comes from a few independent, usually short-term, and research-based endeavors [9]. In the absence of reliable information, we can only obtain a partial picture of what is happening to fish stocks and what could be the best management actions to avoid resource and subsequent societal collapse, as the SSFs are mostly concentrated in the poorest segments of societies [10].

Therefore, in the absence of data, researchers have increasingly used information provided by fishers [11] [12] to fulfill both long- and short-term data gaps [13] [14] [15] [16] [17]. However, although fishers' knowledge can provide detailed information and helpful material to management, especially when there is no other data available [18], it is often difficult to contrast such information with official data. Such comparisons could identify if all or parts of the information provided by fishers were comparable to what would be obtained through formal methods. Fishers hold facts and memories that may not be registered elsewhere [13]; therefore, an understanding of the accuracy of this information could define what can and cannot be applied to management in the absence of better data or as a complement to it. Further, this understanding could avoid costly data sampling procedures [19] and could complement information over temporal scales [20] [21].

Here, we contrasted two formal datasets (governmental and scientific) with data provided by fishers to assess the agreement between the different sources. Specifically, we tested if fishers' perception of past and current catches per unit effort (CPUE) (mean and maximum catches) and rank of species abundance in the catches matched the formal landing data. Understanding the aspects where information agrees and disagrees could show that not all information provided by fishers is the same, which could direct future research addressing unreported but sometimes valuable past and present information to direct management.

## Materials and Methods

### Sampling site

**Fig 1:**
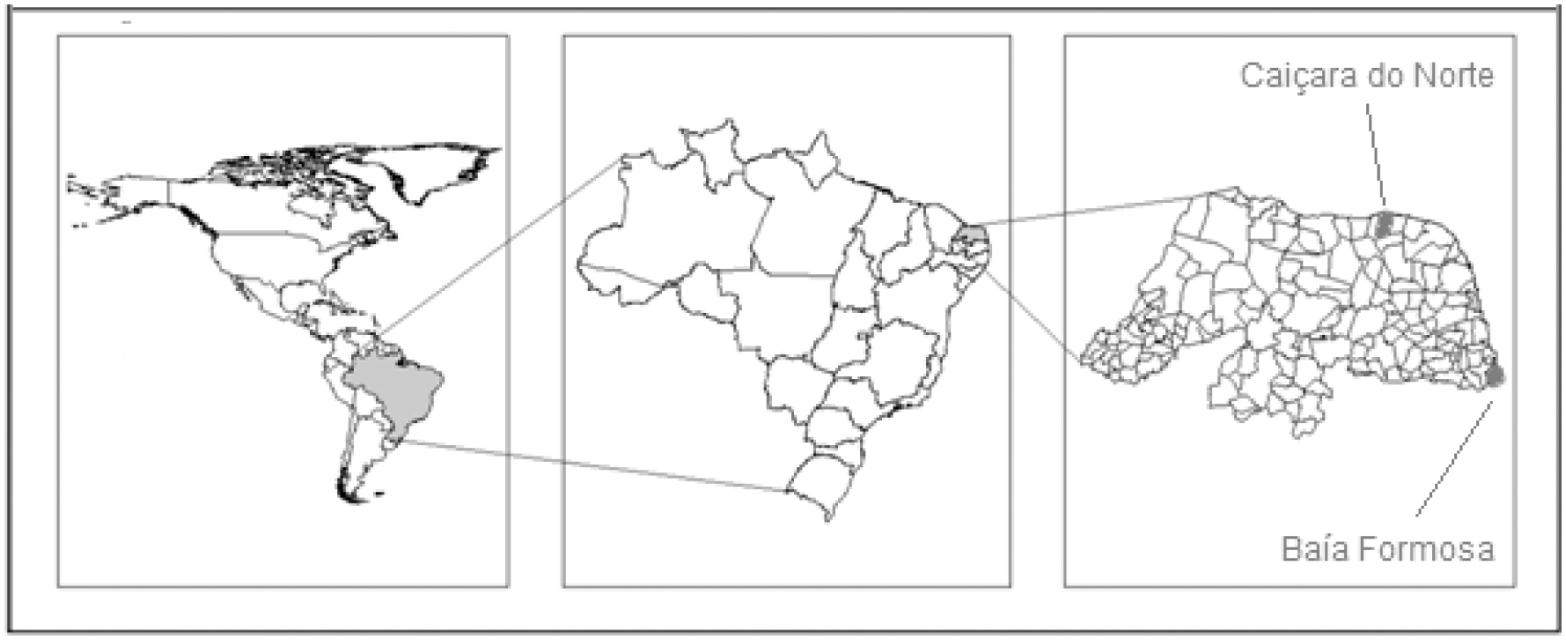
Study area. Fishing communities sampled in the north (Caiçara do Norte) and in the south (Baía Formosa) of the Rio Grande do Norte State, Brazilian northeastern region.

The coastal fishing area assessed is located on the Brazilian northeastern region in the Rio Grande do Norte state (4°49'53''S, 35°58'03''W, and 6°58'57'', 38°36'12''W; Fig 1). This coastline has 25 towns and 93 fishing communities along 399 km. Most of the catch of this state is landed in the capital, Natal (34% of the total landings of the state, mostly industrial). However, the origin of these catches is difficult to trace, as Natal harbors boats with more autonomy, allowing fishing essentially anywhere on the coast and offshore. For these reasons and the impossibility of disaggregating small from large-scale data in Natal, interviews were conducted at two of the main small-scale fishing ports in their respective regions: one north of Natal (Caiçara do Norte—6.9% of all the state landings, herein *North*) and one in the south (Baía Formosa—2.3% of all landings, herein *South*) [22]. Current (scientific sampling) and past official fish landing data were also registered in these two municipalities (Fig 1).

Caiçara do Norte is the largest fishing community northern of Natal, housing 800 active fishers. Since there is no pier or harbor, fishers use to land anywhere along the only beach (8 km long) in the municipality. Because there is no communal landing site and because of the extent of the beach and the history of drug-associated violence in parts of it, the formal sampling effort was concentrated on a third of the area, where roughly 250 fishers land their catches (Colônia de Pescadores, pers. com.). Fortunately, based on what was observed during fieldwork and talks with fishers, this apparently happens to be the place where most fish landings take place. In Caiçara do Norte, the main target fishes are *Coryphaena hippurus* (dolphinfish, locally named *dourado*) and *Hirundichthys affinis* (flying fish, locally named *peixe voador*) [23].

Baía Formosa has a landing site, the Harbor Beach, where all the formal landings were sampled. Baía Formosa's main catches are *Thunnus atlanticus* (blackfin tuna, locally known as *albacora*) and *Lutjanus analis* (mutton snapper, locally known as *cioba*) [23]. In 2014, the local fishers' organization estimated 188 active fishers in Baía Formosa.

As it is common in Brazil, the number of registered fishers considered active by the local fishers’ association does not accurately represent the number of fishers really engaged in fishing. Although some of these fishers will fish only sporadically, they will still be registered to have access to social benefits, such as retirement, pensions and/or a minimum wage during specific fish closed season.

### Scientific, governmental, and informal data assessed

#### Scientific and governmental data

A research team recorded fish landing (hereafter referred to as scientific data) from January 2013 to March 2014, an interval chosen for including a full fishing cycle, from the blackfin tuna period to black grouper (*Mycteroperca bonaci*) migration period. Both ports (in the north and south) were visited simultaneously on two days each month. All landings from 6 am to 6 pm were registered. Information on the amount of species caught, fishing effort, and fishing gear (types and quantities) were recorded. Fishing effort was measured as hours at sea and crew size.

The nation's environmental agency, The Brazilian Institute of Environment and Renewable Natural Resources (IBAMA), provided past landing data for the two studied areas only for 2003, because neither IBAMA nor any other governmental body sampled any data for 1993 or 2013. Both the governmental and the scientific data collection followed the same interview protocol. However, the quality of the governmental data, which was used to inform the Food and Agriculture Organization (FAO), has been questioned before, as have data provided by multiple countries [24].

#### Informal data to reconstruct catches and fish abundance

The research team also gathered informal data through face-to-face interviews with 82 fishers (North = 50; South = 32) and nine expert fishers (North = 5; South = 4). As explained earlier, although many fishers are considered officially active, they are in fact “registered” fishers, fishing rarely. These sporadic fishers were not interviewed, as it was expected that they would not be able to provide detailed past information. After providing personal information, such as name, age, and fishing experience, fishers were asked questions that would allow the reconstruction of catches and the establishment of a rank of abundance of fish species based on what they see in the catches for 2013 (the time of the interview), 2003, and 1993. Such a rank of abundance based on catches does not intend to accurately reflect biological abundance. Only fishers older than 18 years and with more than five years of fishing experience were interviewed. If the fisher had less than 20 years of experience, he was interviewed only for the reconstruction of CPUE and to inform the rank of species abundance for the fishery in 2013. The interviewees' fishing experience ranged from 20 to 62 years (in the South) and 23 to 60 years (in the North), and their average age was 43,7 and 43 years old, respectively.

Data sampling was approved according to the guidelines of the Committee of Ethics at the Federal University of Rio Grande do Norte (Protocol of approval No.19685113.3.0000.5537). Before each interview, fishers were approached and informed about the research purpose. Only those who gave verbal consent were included in the study. The ethical committees’ guidelines allow oral consent due to the likelihood of illiterate interviewees and/or traditional communities not willing to tape recording. Here the verbal consent was indeed necessary due to the high proportion of illiteracy among fishers and was always witnessed by a third person from the university but not involved in the research.

#### Data sampling to reconstruct catches

The interviewed fishers provided information on crew sizes, hours at sea, catches, and type and number of gear used. As in other studies recording fishers' memories (e.g., [7]), a few questions were adapted to guarantee better understanding by fishers and to elicit their memories. Accordingly, for each of the three years (2013, 2003, and 1993), fishers were asked about the fish species they caught and catches per species (kg) in their best catches. For each of the species cited, they were asked to estimate the average catches for each given year. However, at first the fishers did not understand that a mean catch meant their regular catches. Thus, they were asked about those catches they considered neither good enough to be called great catches nor bad enough to be considered among the worst. The fishers understood this concept and were able to estimate mean catches informing species and amount caught.

To avoid the tendency of fishers to believe that past catches were always better than current catches (called retrospective bias by [25]), the fishers were not asked to provide information chronologically, but were asked first about 2003, then 1993, and, finally, 2013.

#### Data sampling to reconstruct fish abundance

For 2003, an estimate of abundance of species, as seen in the landings (in kg), was extracted from the governmental data supplied by IBAMA. Although the expression abundance is used throughout the text, it should be carefully interpreted, as landing data does not necessarily reflect biological abundance [2]. Such estimate was compared with a rank of species abundance established by the fishers themselves, who most likely also take into account what they see in their catches. These species from the governmental data were categorized as high, average, and low abundance, and the intervals for each of the categories varied according to the municipality. This was necessary because the municipalities tend to focus on different species with very discrepant abundances.

In the southern community, the intervals were coded following a logarithmic scale [26]: species that represented more than 7t in the official records for 2003 were considered highly abundant; species with total catches between 0.3t and 2t were classified as of average abundance (there were no species between 2.1 and 6.9t); and any species below that level were classified as low abundance. Twenty species were chosen from the final list containing high-, average-, and low abundance species to be discussed in interviews (four, three and four, respectively). Fishers were presented pictures of these 11 species and asked to rank them from the highest to the lowest abundances in current catches and in catches from 10 and 20 years past.

However, this approach did not work in the North. Fishers simply did not understand how to rank the species shown in the pictures. Therefore, they were asked to cite species of high-, average-, and low-abundance for 2013, for 10 years, and 20 years past, regardless of the pictures. In both areas (North and South) the fishers spontaneously informed when the low abundance species became scarce or completely disappeared.

#### Fish species abundance reconstructed by expert fishers

Nine fishers (five in the South and four in the North) were approached as expert fishers (hereafter EFs), following multiple suggestions of currently active fishers. EFs are older and more experienced fishers who are expected to have specific and detailed knowledge on local fishery issues. Here, EFs included those with over 40 years experience in fishing and who had lived their entire lives in the same communities; all of the EFs had stopped fishing but still visited the landing sites daily, mainly because of their influential status as knowledgeable fishers.

The EFs were asked, through face-to-face interviews, which species were most common in the catches when they began to fish, which species decreased or disappeared from the catches since then, and whether any other species had become fishing targets since they started fishing.

The EFs were assessed to contrast information provided by regular fishers regarding the fishing status of 20 years ago (target species, abundance, and CPUE) because this information was not available elsewhere.

#### CPUE estimates

The CPUE was defined as catches (kg)/number of fishers × hours fishing; and was calculated for the best catches (maximum CPUE) and for average catches (mean CPUE) using official and informal (interviews) datasets. The fishers' data provided information to calculate CPUE for 1993, 2003, and 2013. However, since the EFs were the only source of information for 1993 and they were not able to provide CPUE data for 1993, the fishers’ CPUE could only be compared to: 1) governmental data for 2003 (fish landing sampled by IBAMA); and 2) scientific data (fish landing sampled by researchers) for 2013. Therefore, the CPUE estimated by fishers for 1993 is not shown here.

The CPUE was estimated per species and per fishing gear. Only species registered in at least two landing samplings and mentioned by at least two fishers during the interviews were used to calculate the estimates. The modal value for the variable hours at sea and crew size, per gear, was used to fill in the occasional lack of information in the governmental data for 2003.

#### Questions

**Fig 2:**
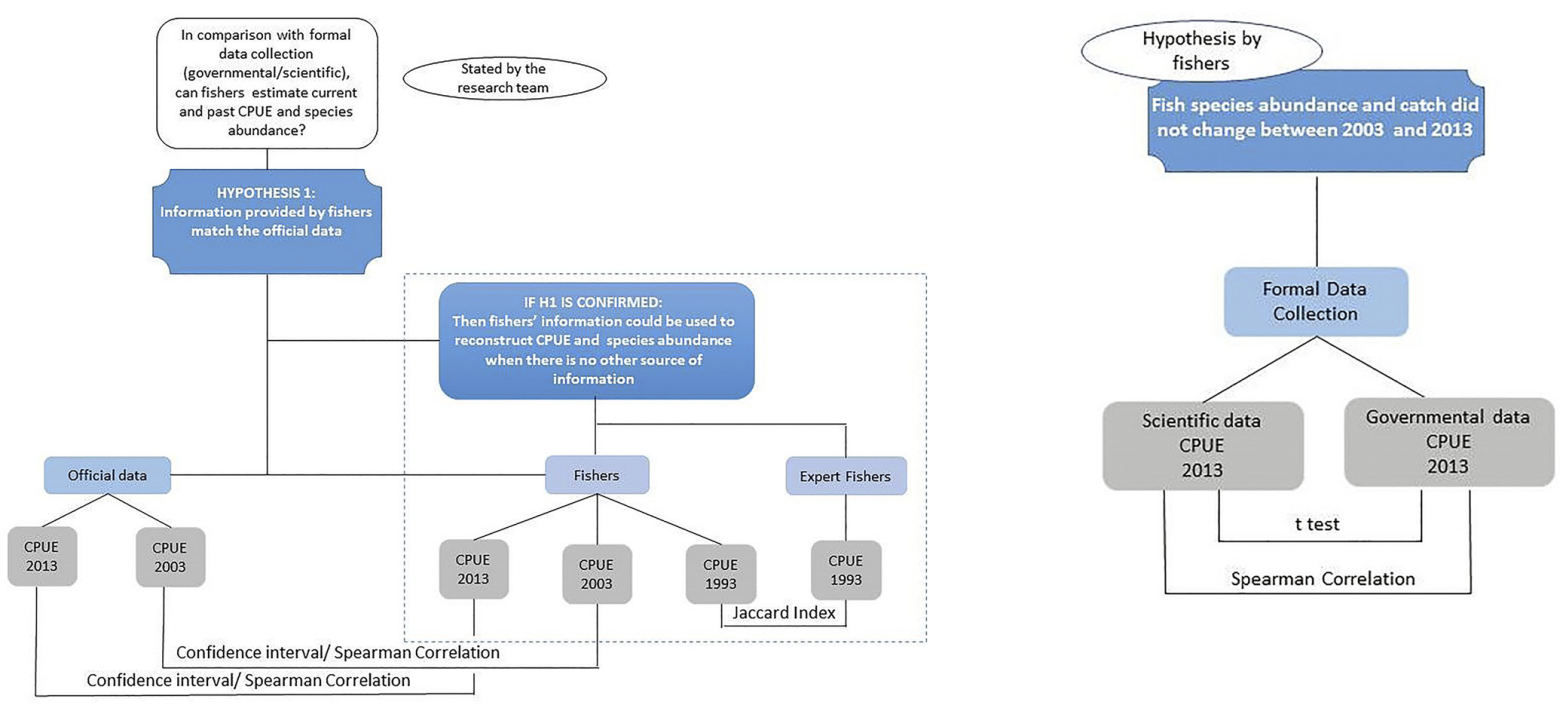
Research outline. Questions and hypotheses raised by the research team and fishers that guided the statistical analyses performed regarding the CPUE for 1993, 2003, and 2013. (See text for details.)

Two different questions related to catches were statistically tested, as detailed in Fig 2.

1. Did the fisheries change between 2003 and 2013? In the interviews, all fishers claimed that the CPUE and rank of species abundance did not change between 2003 and 2013. To investigate if fishers were correct in their collective belief, the CPUE from governmental data (2003) and the CPUE from scientific data (2013) were compared by t-test using GraphPad Prism Version 6.01 for Windows. The data from the South had to be bootstrapped due to data heteroscedasticity. To test fishers' perceptions about the lack of change in the rank of species abundance, all species were first ranked for both years using the official data sources (governmental—2003, and scientific—2013), from the most to the least abundant in the sampled catches. The two official ranks were then compared by the Spearman's rank correlation coefficient. This non-parametric test measured the strength of the association between two ordinal variables using the order in which the species appeared rather than the observed values.
2. Can fishers provide accurate estimates for past, present, and maximum CPUE and for changes in the rank of species abundance? A confidence interval analysis was performed to assess the difference between the CPUE by species reconstructed by fishers and the CPUE estimated from the governmental data (2003) and the scientific landing sampling (2013). The difference between the CPUE estimated using the data provided by fishers and the governmental/scientific data were bootstrapped using Microsoft Excel and the supplement Pop Tools [27]. Negative confidence interval values meant that fishers underestimated the CPUE for a given species, while values higher than zero meant that fishers overestimated the species' CPUE. Values crossing the zero line showed no difference between the CPUE estimated by fishers and that provided by governmental/scientific data.

Fishers provided information that allowed the estimation of 1,075 individual CPUE for 43 species caught in 2003 or in 2013. However, for better accuracy, only species quoted by two fishers and also recorded in the governmental and in the scientific sampling had their CPUE taken into account, which resulted in 763 individual CPUE for 22 species. A list of species registered in the landing samplings and mentioned by the interviewees is provided in S1 Appendix.

For 1993, the information on the rank of species abundance provided by fishers was compared to information provided by EFs on species that they considered abundant and species they thought had decreased or disappeared from the catches, which was used as a control, assuming that the experts had better knowledge. In this case, as the information was number of citations per species, the comparison was done using the Jaccard similarity coefficient (Fig 2).

The Spearman's rank correlation coefficient was used to verify whether the rank of species abundance reported by fishers for 2003 and 2013 in the South and North areas corresponded to the species that comprised most of landings (in kg), in the same order, in the governmental and scientific data for the same years respectively.

## Results

### Reconstructing catches

**Fig 3:**
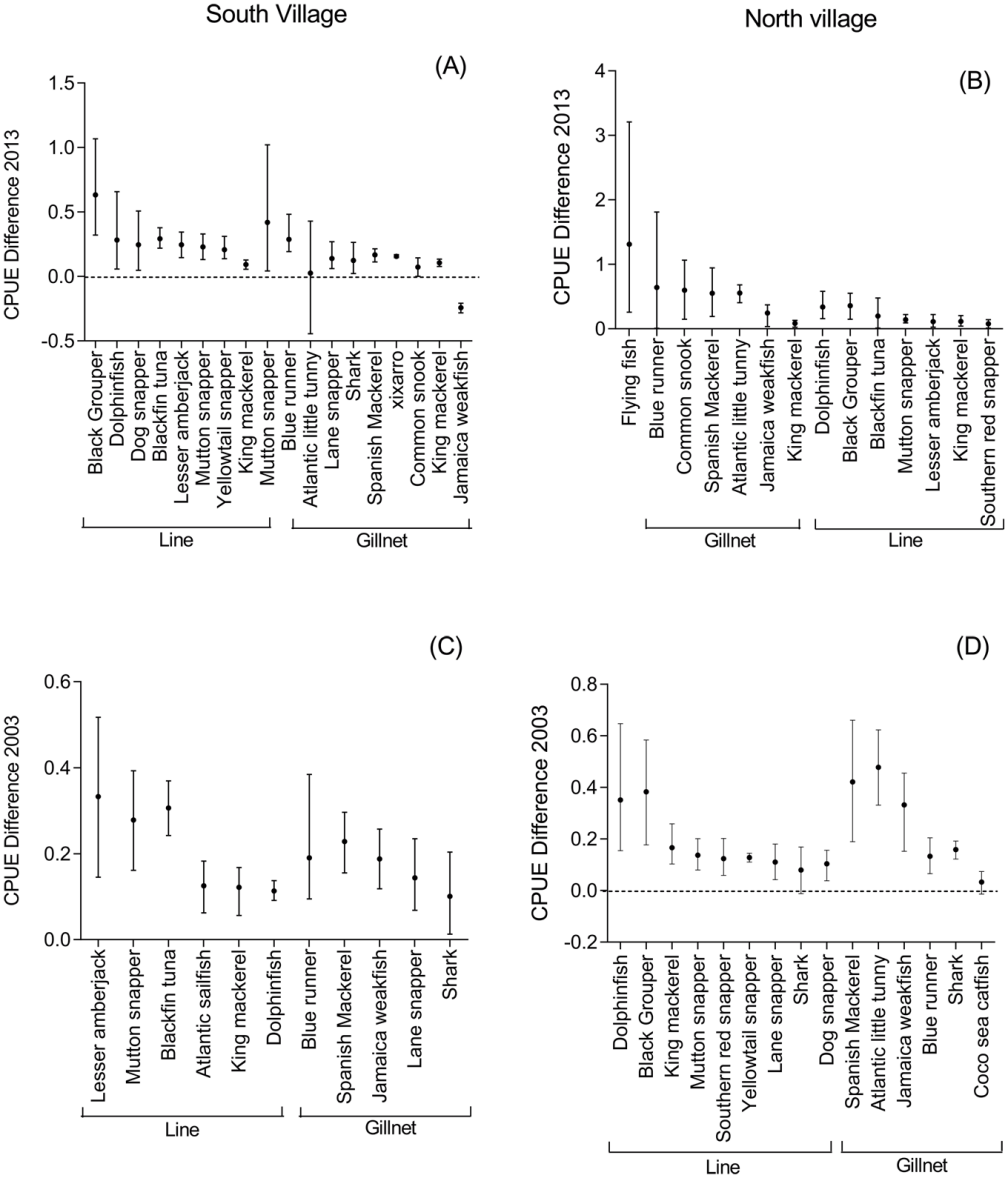
Difference between mean CPUE informed by fishers and mean CPUE of formal data. Mean value and confidence interval of the difference between the CPUE provided by fishers (2003 and 2013) and the CPUE calculated from governmental data (2003) and from scientific data (2013). Fig A: South municipality in 2013; Fig B: North municipality in 2013; Fig C: South municipality in 2003, and Fig D: North municipality in 2003. Flying fish was not included due to its large confidence interval (CI—upper: 2.64; lower: 0.23). Dotted line is not shown when the difference between the values was not negative (as in Fig 3B and 3C).

1. Did the fisheries change between 2003 and 2013? All fishers said that their fisheries did not change between 2003 and 2013. Confirming their claim, the CPUE estimated from the governmental landing data (for 2003) did not differ from the CPUE estimated from the scientific landing data (2013) in both municipalities (South: t = 0.1, p = 0.202; North: t = 1.6, p = 0.118).
  Conversely, the species abundance rank differed between 2003 (governmental data) and 2013 (scientific data) for the South (Spearman's r = 0.38, p = 0.15), but not for the North (Spearman's r = 0.6, p = 0.003).
2. Can fishers provide accurate estimates for mean past, mean present, and maximum CPUE and for changes in the rank of species abundance? Fishers tended to overestimate the mean CPUE for roughly all species, regardless of the year or gear used to exploit each species. The fishers' information only matched official data significantly for three fish species/group of species caught by gillnets: Atlantic little tunny (Fig 3A), sharks, and coco sea catfish (Fig 3D). For the other 13 species, fishers' information was close but not statistically equal to official data (Fig 3).

**Fig 4:**
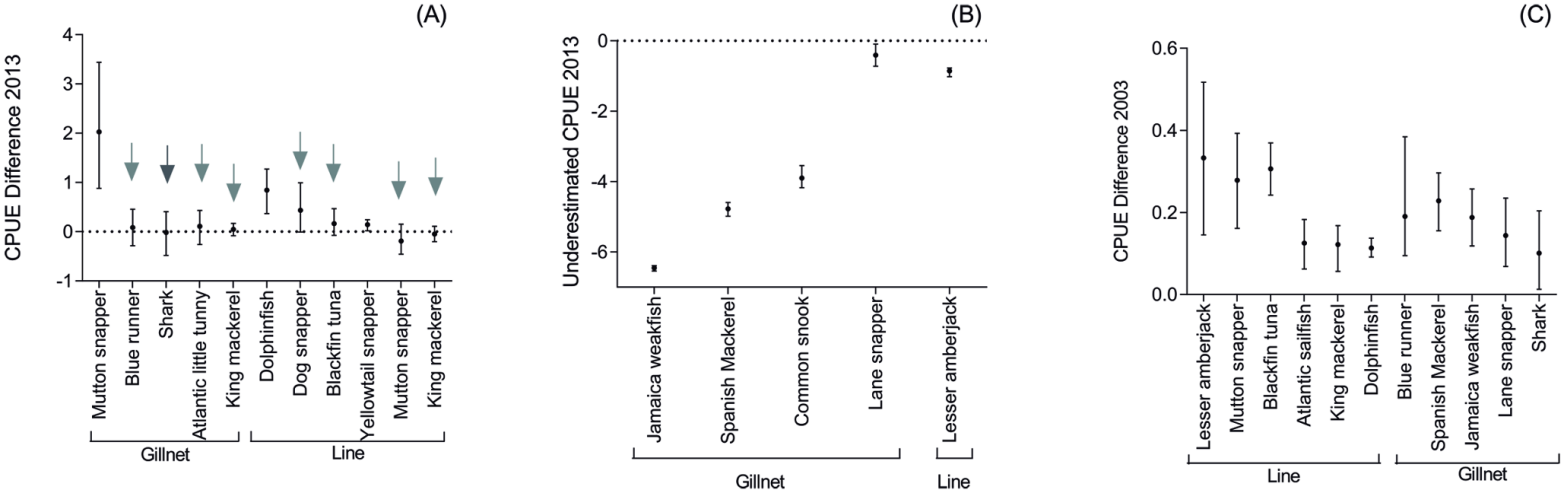
Difference between maximum CPUE informed by fishers and maximum CPUE of scientific data. Mean value and confidence interval of the difference between maximum CPUE according to fishers and according to scientific landing data (records for 2013). (A) CPUE overestimated (vertical dotted line) by fishers or similar to the official data (see arrows) in the South municipality; (B) CPUE underestimated by fishers in the South municipality. Black Grouper (underestimated) is not shown due to its large confidence interval (mean: -10.489; upper: -9.954; lower: -10.934); (C) CPUE overestimated or similar (arrows) to the CPUE of official data in the North municipality.

The information provided by fishers on their maximum CPUE allowed the calculation of 55 CPUE estimated for the years 2003 and 2013 to 18 species caught by handline, gillnet or *jereré*. These were compared to the maximum CPUE registered in the governmental (2003) and scientific (2013) data. Of the 55 compared CPUE, 18 were not statistically different (the significant measures are identified by arrows in Fig 4). However, of these 18, 16 (eight in each region) referred to 2013 mostly from fishing done with handlines. The remaining measures were usually overestimates of the fishers' maximum CPUE.

**Fig 5:**
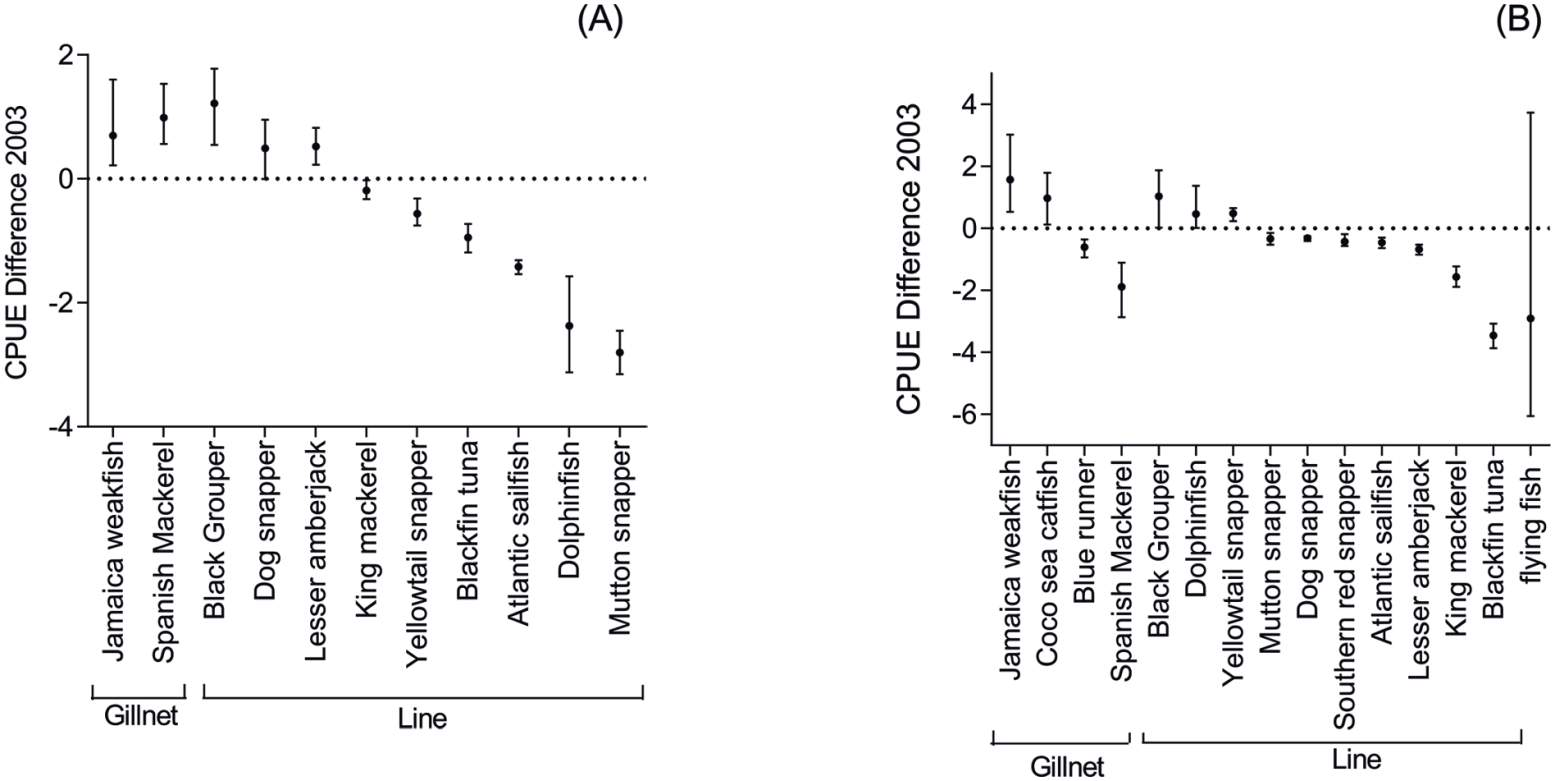
Difference between maximum CPUE informed by fishers and maximum CPUE of governmental data. Mean value and confidence interval of the difference between the maximum CPUE estimated using fishers' information and the maximum CPUE according to the governmental data (landing records for 2003) in the South municipality (A) and the North municipality (B).

For 2003, fishers' information on their maximum CPUE only matched the maximum CPUE registered in the governmental data for dog snapper (Fig 5A) and flying fish (Fig 5B).

**Fig 6:**
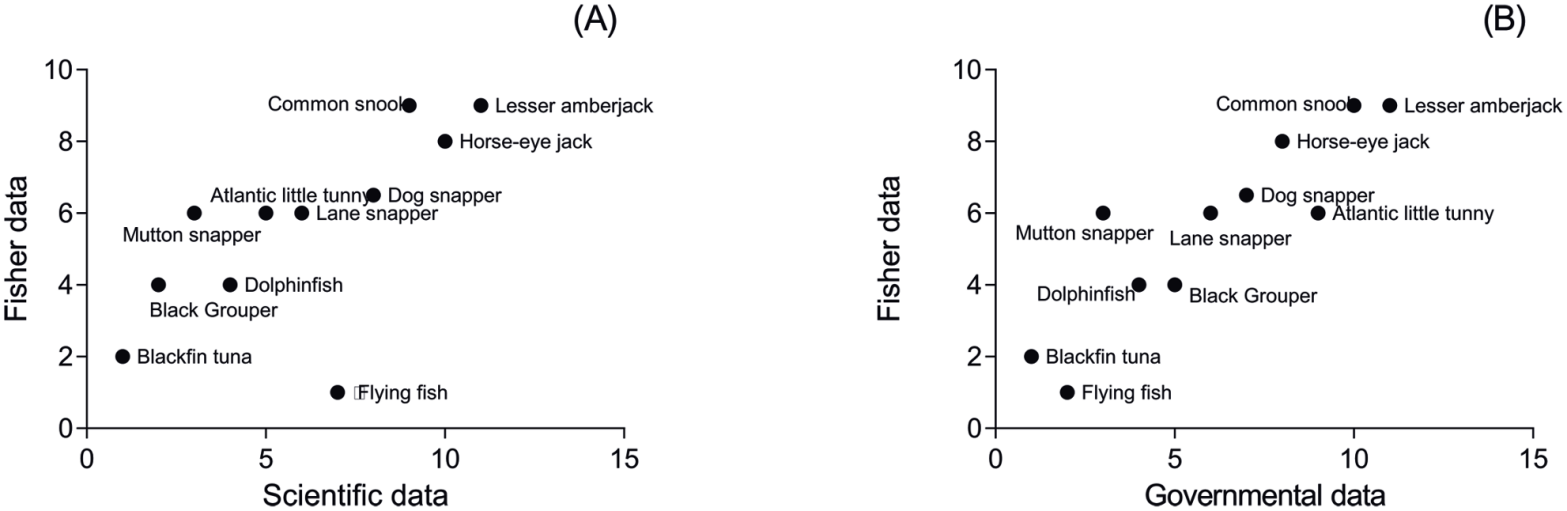
Correlation of the rank of species abundance, based on catch data, according to fishers from the South with the rank of species abundance, also based on catch data, according to formal data. (A) Scientific landing data for 2013 (r = 0.88, p = 0.000) and (B) Governmental landing data for 2003 (r = 0.75, p = 0.009).

The rank of species abundance provided by fishers from the South was highly correlated with the rank of species abundance estimated from the governmental data for 2003 (r = 0.75; p = 0.009) and with the rank of species abundance estimated from the scientific data for 2013 (r = 0.88; p = 0.000; Fig 6). The correlations for the North were also significant (2003: r = 0.4, p = 0.01; 2013: r = 0.3, p = 0.05).

The EFs reported the decrease or disappearance of 10 species from the fish landings in the last 40 years. In fact, four species mentioned by the EFs were not recorded in the landings for 2013 or 2003 (northern red snapper, Atlantic goliath grouper, and two species of Atlantic bumper). For the other four species (lesser amberjack, black salmon, great hammerhead, and queen triggerfish), the EFs reported low catches since 2003. Even though the EFs pointed out decreased catches of dolphinfish and Spanish mackerel, these species are still among those most caught in the southern and northern coastal areas.

Fishers were able to provide more information on their maximum CPUE than on their mean CPUE for 1993, which allowed estimates of 77 CPUE of maximum CPUE and 45 mean CPUE of average catches. However, this information could not be compared to other measures due to lack of any source of official data and difficulties the EFs had reporting quantitative data.

Both in the South (Jaccard index: 0.33) and North (Jaccard index: 0.37), there were moderate similarities in the species composition for 1993 between the information provided by fishers and the information provided by EFs.

## Discussion

Since Johannes' seminal paper in 1981 [28], which suggested the importance of using fishers' knowledge, different studies have supported his claims, showing the consistency of data provided by fishers with direct use in fishery management [7] [21] [11]. Recently, some researches have shown that fishers' knowledge, if combined with scientific data, could also be useful in filling the gaps required to understand fisheries [17] [13]. Here, official data were compared to information provided by fishers regarding CPUE and target species. Additionally, one hypothesis raised by the fishers themselves (i.e., that the CPUE did not change in the last decade) was tested using only official data. If proved correct, this last hypothesis could also give support to fishers' knowledge. Indeed, as claimed by fishers, the findings did not show any change in the CPUE from 2003 to 2013. In addition to being good informants to establish ranks of abundance for their target species, as perceived from the catches, fishers also provided accurate estimates of their maximum CPUE in the most recent period (2013).

However, fishers tended to overestimate mean CPUE. Some research has suggested that fishers can have trouble perceiving trends in catch variability along time series [29], which could make it difficult to measure this variability in relation to fishing effort. This could explain the difficulties fishers had when informing CPUE. On the other hand, some researches have suggested that official data, mostly governmental data provided to FAO, usually underestimates catches, mainly in small-scale fisheries [30] [31] [32]. Therefore, at least in 2003, fishers' information cannot be completely disregarded. Such discrepancies between the data sources has yet another implication. While management is based on official data sources, fishers will keep believing in their perceptions of higher values of CPUE, threatening the success of any management actions due to lack of compliance [33]. Thus, assessing the agreement between governmental and scientific CPUE with the CPUE perceived by fishers can guide managers on how to address the lack of acceptance among fishers, who do not understand why such measures are necessary if fisheries, according to their beliefs, have not changed.

The fact that the CPUE did not change between 2003 and 2013 does not necessarily imply no changes in the fish stock. Multiple factors that mask changes in CPUE could have happened, such as increase in the proportions and sizes (for nets) of gears used, area expansion [34], and range collapse, when decreasing populations of school fish rearrange in fewer but constant size schools, keeping the catches at similar levels for a while, until the stock collapses [35]. This can also be a source of confusion for fishers, as they may not perceive changes in their catches, in time spent fishing, and, consequently, in stocks. This also means that for some fishers, especially the younger ones, this may suggest productive stocks, which disagrees with the opinion of expert fishers. Experts indicated lower catches for multiple species, including those more commonly caught currently, such as dolphinfish and Spanish mackerel. The different views between older and younger fishers qualify for what the literature refers to as the shifting baseline syndrome [36]. Similarly, some evidence of the shifting baseline syndrome can also be seen when younger fishers did not cite the same species formerly caught by EFs (see Jaccard index). Further studies could confirm if this is a general pattern, and if so, EFs could help identify not only depletion, but also local extinctions that younger fishers may not be aware of.

The good precision of fishers when reporting their maximum CPUE has been registered recently in other studies (e.g., [7]). Fishers may better remember their best catches because such memories are associated with uncommon and pleasant moments [37]. However, it is worth noting that, here, their memories of maximum CPUE and the most abundant species referred mainly to migratory target fishes. Therefore, when attempting to reconstruct data from fishers' recollections, it may be important to notice if a species is a main target. Fishers may be less accurate the less important a species is for their economy.

The approach used is this study showed, once again, that fishers hold important and sometimes unique information on fisheries issues. However, different than other studies, when such knowledge was compared to more formal data, it also showed important divergences, mainly suggesting that fishers tend to overestimate their CPUE and are more accurate in reporting CPUE on recent best catches. This should be seen as a caution sign when using fishers' information: fishers, just like everybody else, may forget or mix information. Finally, the use of official data combined with information provided by expert fishers allowed the reconstruction of CPUE for two periods using two different sources of data. This heterogeneity of informants and data sources may be the only and best source of information for fisheries in areas where there is shortage of scientific data but abundance of skillful fishers.

## Supporting Information

S1 Appendix List of the species registered in landings and/or mentioned by the interviewees. (DOCX)
