# Supplementary material for: Matching Fishers’ Knowledge and Landing Data to Overcome Data Missing in Small-Scale Fisheries
Source: PLoS One. 2015 Jul 15;10(7):e0133122. doi: 10.1371/journal.pone.0133122 (PMC4503677; doi:10.1371/journal.pone.0133122)
Supplement: S1 Appendix — (DOCX) [file pone.0133122.s001.docx]

| Common name - Portuguese | Scientific name | Common name - English |
| --- | --- | --- |
| Agulha | *Hemirhamphus brasiliensis* | Ballyhoo |
| Agulhão chato |  |  |
| Agulhão de vela/Agulhão | *Istiophorus albicans* | Atlantic sailfish |
| Albacora | *Thunnus atlanticus* | Blackfin tuna |
| Arabaiana | *Seriola fasciata* | Lesser amberjack |
| Arabaiana chata | *Seriola rivoliana* | Longfin yellowtail |
| Ariacó | *Lutjanus synagris* | Lane snapper |
| Bagre | *Bagre bagre* | Coco sea catfish |
| Bejupirá | *Rachycentron canadum* | Black salmon |
| Boca mole | *Larimus breviceps* | Silver snapper |
| Bonito | *Euthynnus alletteratus* | Atlantic little tunny |
| Cação | Many sharks species from different families | Shark |
| Cação cavala | *Isurus oxyrinchus* | Shortfin mako shark |
| Cambuba | *Haemulon parra* | Sailor's grunt |
| Camurim | *Centropomus undecimalis* | Common snook |
| Camurupim | *Megalops atlanticus* | Tarpon |
| Cavala branca/Cavala | *Scomberomorus cavalla* | King mackerel |
| Cavala preta/Cavala | *Acanthocybium solandri* | Wahoo |
| Cioba | *Lutjanus analis* | Mutton snapper |
| Corá | *Lutjanus campechanus* | Northern red snapper |
| Cururuca | *Micropogonias furnieri* | Whitemouth croaker |
| Curvina | *Pareques acuminatus* | High-hat |
| Dentão | *Lutjanus jocu* | Dog snapper |
| Dorminhoco | *Lobotes surinamensis* | Tripletail |
| Dourado | *Coryphaena hippurus* | Dolphinfish |
| Espada | *Trichiurus lepturus* | Largehead hairtail |
| Galo do alto | *Alectis ciliaris* | African pompano |
| Garacimbora | *Caranx latus* | Horse-eye jack |
| Garajuba/ Garaxumba | *Caranx crysos* | Blue runner |
| Guaiuba | *Ocyurus chrysurus* | Yellowtail snapper |
| Judeu | *Menticirrhus americanus* | Southern kingcroaker |
| Mero | *Epinephelus itajara* | Atlantic goliath grouper |
| Palombeta açu/Palombeta chata | *Chloroscombrus chrysurus* | Atlantic bumper |
| Pargo | *Lutjanus purpureus* | Southern red snapper |
| Pescada | *Cynoscion jamaicensis* | Jamaica weakfish |
| Salema | *Anisotremus virginicus* | Porkfish |
| Sardinha | *Opisthonema oglinun* | [Atlantic thread herring](http://www.fishbase.org/ComNames/CommonNameSummary.php?autoctr=3308) |
| Serra | *Scomberomorus brasiliensis* | Spanish Mackerel |
| Sirigado | *Mycteroperca bonaci* | Black Grouper |
| Tainha | *Mugil curema* | White mullet |
| Voador | *Hirundichthys affins* | Flying fish |
| Xaréu | *Caranx lugubris* | Black Jack |
| Xixarro | *Decapterus punctatus* | Round scad |
